# Sex-biased immunological processes drive hidradenitis suppurativa

**DOI:** 10.3389/fimmu.2023.1167021

**Published:** 2023-05-04

**Authors:** Kelly Z. Young, Peter Dimitrion, Li Zhou, Indra Adrianto, Qing-Sheng Mi

**Affiliations:** ^1^ Center for Cutaneous Biology and Immunology Research, Department of Dermatology, Henry Ford Health, Detroit, MI, United States; ^2^ University of Michigan Medical School, Ann Arbor, MI, United States; ^3^ Immunology Research Program, Henry Ford Cancer Institute, Henry Ford Health, Detroit, MI, United States; ^4^ Cancer Biology Graduate Program, School of Medicine, Wayne State University, Detroit, MI, United States; ^5^ Department of Medicine, College of Human Medicine, Michigan State University, East Lansing, MI, United States; ^6^ Center for Bioinformatics, Department of Public Health Sciences, Henry Ford Health, Detroit, MI, United States

**Keywords:** hidradenitis suppurativa, sex, immune activation, Th17, hormones, X chromosome

## Abstract

Hidradenitis suppurativa (HS) is a chronic inflammatory skin condition that can manifest with abscesses, sinus tracts, and scarring in the intertriginous areas of the body. HS is characterized by immune dysregulation, featuring elevated levels of myeloid cells, T helper (Th) cells, and pro-inflammatory cytokines, particularly those involved in Th1- and Th17-mediated immunity. In most epidemiological studies, HS shows a strong female sex bias, with reported female-to-male ratios estimated at roughly 3:1, suggesting that sex-related factors contribute to HS pathophysiology. In this article, we review the role of intrinsic and extrinsic factors that contribute to immunological differences between the sexes and postulate their role in the female sex bias observed in HS. We discuss the effects of hormones, X chromosome dosage, genetics, the microbiome, and smoking on sex-related differences in immunity to postulate potential immunological mechanisms in HS pathophysiology. Future studies are required to better characterize sex-biased factors that contribute to HS disease presentations.

## 1 Introduction

Hidradenitis suppurativa (HS) is a chronic inflammatory skin condition clinically characterized by abscesses, sinus tracts, and scarring that commonly affects intertriginous skin (1). Although thought to be a relatively rare disorder, with reported prevalence rates ranging from 0.00033% to 4.1% (2), HS carries a significant disease burden, as it is associated with dramatic impairment in patient quality of life and increased rates of health comorbidities (1). Currently, the mechanisms underlying HS remain unclear, and an improved understanding of disease pathogenesis is imperative for the development of new therapies.

**Figure 1:**
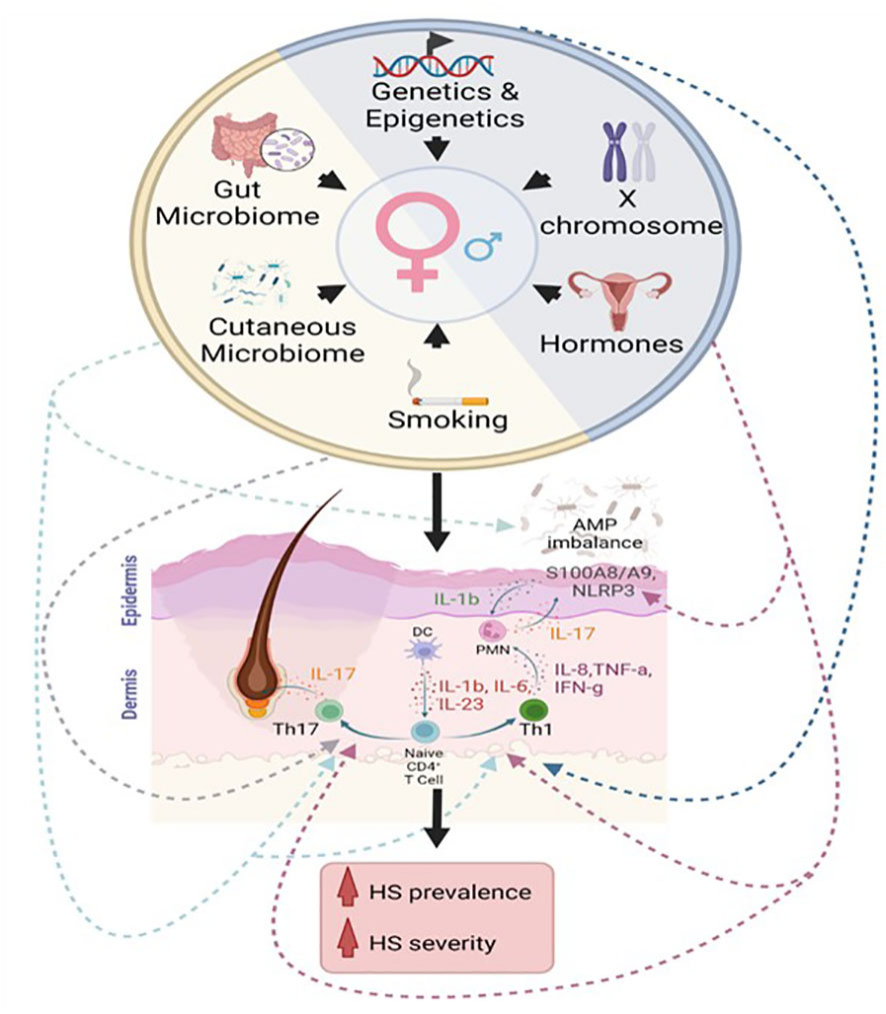
Proposed immunological contributions to the female sex bias in hidradenitis suppurativa. Intrinsic factors (blue semicircle) include genetic, epigenetic, hormonal, and chromosomal contributions to sex-related differences in immunity. Extrinsic factors (yellow semicircle) include contributions from the cutaneous microbiome, gut microbiome, and environmental influences, such as smoking. We propose that these factors affect the Th1 pathway, Th17 pathway, inflammasome activation, and AMP dysregulation in hidradenitis suppurativa.

Patients with HS exhibit marked cutaneous immune dysregulation, featuring elevated levels of innate immune cells, adaptive immune cells, and pro-inflammatory cytokines, particularly those involved in Th1 and Th17 pathways, such as interleukin (IL)17, IL6, IL23, IL1β, IL12, interferon (IFN)-γ, IL8, and tumor necrosis factor (TNF)-alpha (**Figure 1**) (3–5). The clinical efficacy of cytokine blocking immunotherapy—such as adalimumab, secukinumab, and brodalumab—supports a role for inflammatory cytokines as key mediators of disease (3, 6–9). For example, brodalumab, which is a monoclonal antibody that inhibits IL-17 receptor A, has been shown to reduce the level of circulating cutaneous inflammatory cytokines known to promote neutrophil, T cell, and B cells, mediated inflammatory processes (10). Additionally, altered levels of antimicrobial peptides (AMPs), which are important elements of innate immunity and potent immunomodulators, are often seen in lesional and nonlesional skin in patients with HS (**Figure 1**) (4, 11).

Geographic variability is evident in the epidemiologic data on HS, but most studies from North America and Europe report a female-to-male ratio of ~3:1 (12). This female sex bias is reminiscent of several autoimmune conditions, such as Sjögren syndrome (SS), systemic sclerosis (SSc), and systemic lupus erythematosus (SLE), which also feature a dramatic female sex bias (13). Although HS is thought to have more of an autoinflammatory rather than autoimmune component, similar processes may contribute to the female sex bias in HS. Through a review of both intrinsic and extrinsic sex-biased immunological contributions to select autoimmune disorders, we discuss the potential mechanisms that may contribute to the female sex bias observed in HS. Note: In this manuscript, “sex” will be used to denote biologic differences in sex chromosomes, hormones, and gonads, while “gender” takes into account psychosocial and cultural factors (12–14).

## 2 Intrinsic immunologic contributions to the increased prevalence of hidradenitis suppurativa in biologic females

How biological sex influences the immune system has recently been reviewed by Jiwrajka and Anguera (14). The idea that biological females exhibit greater thymic output than biological males is becoming increasingly accepted, partially explaining the larger proportion of T cells that comprise the adult female immune system. Females also harbor a greater proportion of B cells, whereas males are known to have greater proportions of natural killer cells and monocytes (14). Broadly speaking, innate, antigen-specific, and humoral immune responses are more robust in biologic females (13, 15), which may be explained by factors intrinsic to females. While antigen specificity has not been defined as a feature of HS, innate and humoral immune responses are clear features of HS (16, 17), and evidence of the effects of hormones, X-chromosome dosage, and sex-biased genes on immunological pathways highlights potential contributors to the female sex bias in HS.

### 2.1 Hormones

Hormonal fluctuations during puberty, the menstrual cycle, pregnancy, and menopause have been associated with the onset of HS and/or HS flares in some patients, highlighting that the effect of sex hormones on the cutaneous immune system may contribute to HS pathogenesis (18). Hormones regulate immune cell function through nuclear and non-nuclear receptors (15, 19). Studies using animal models have found that females have more robust Th1 adaptive immunity, and naive CD4+ T cells from females proliferate more rapidly and secrete higher levels of IFN-gamma than male T cells (20). Estrogen has complex, varying effects on the female immune response and has been extensively reviewed elsewhere (19). This hormone enhances lymphoid cell proliferation, survival, and cytokine expression—notably IFN-γ (19). At certain concentrations, estrogen can stimulate secretion of IFN-γ and promote a Th1 response (19). Thus, the elevated IFN-γ observed in female patients with HS may result, in part, from the effects of estrogen signaling in lymphoid cells, mostly T and natural killer cells (19).

Numerous studies have also associated HS with acne, hirsutism, and polycystic ovarian syndrome, supporting a role for androgens on disease (21). Furthermore, recent case reports have described new-onset and exacerbation of HS symptoms in gender dysphoric patients undergoing supplemental testosterone therapy (22). Higher proportions of androgen receptor–positive keratinocytes are found in HS lesional skin relative to healthy skin, and microarray studies indicate enrichment of androgen receptor–regulated genes in HS lesional skin (23). Although androgens are generally thought of as having immunosuppressive properties, one potential mechanism contributing to inflammation in HS includes androgen-associated enhancement of Th17 responses resulting in increased IL17 production (19, 20). Notably, some patients report clinical improvement with anti-androgen therapy, including spironolactone, finasteride, and antiandrogenic progesterones (21, 24, 25). While estrogens and androgens may have opposing function in developmental processes (i.e., sex-determination and puberty), these data highlight that their influence on the immune response is more likely due to combined interactions and relative changes in concentrations leading to immune perturbations.

Sex hormones also have effects on cutaneous innate immune responses, one of which is activation of the NLRP3 inflammasome (23, 26), which is important for the process of damage-associated molecular pattern (DAMP) and pathogen-associated molecular pattern (PAMP) recognition (26). The NLRP3 inflammasome activates caspase-1, which leads to the activation of IL1β and IL18 from their pro-peptides, and these molecules have broad effects on downstream immunological activity, such as increasing the expression of chemokines and other inflammatory cytokines (e.g., IL6, TNF-α) (23, 24). In certain noncutaneous disorders, androgens have been suggested to directly activate the NLRP3 inflammasome, which provides an interesting hypothesis that may explain the exacerbated HS seen in transgender men who initiate androgenic gender-affirming therapy (20, 22). Also, activating mutations in *MEFV*, which encodes the pyrin protein of the pyrin inflammasome, have been found in patients with specific syndromic forms of HS, although syndromic HS typically encompasses additional findings, such as osteoarticular manifestations (4, 25–27). Several studies have also identified elevated IL1beta and increased NLRP3 expression in skin lesions of patients with HS (28, 29). Furthermore, HS lesions harbor features suggestive of inflammasome activity, including enhanced caspase-1 activity (28). As such, the NLRP3 inflammasome may play a role in HS, and the effect of sex hormones on NLRP3 activation may further contribute to sex-related differences in HS. As of now, the precise roles for different hormones have not been well placed in HS pathophysiology. Future studies elucidating the effects of sex hormones on inflammasome activation or immune cell function in patients with HS may support a mechanistic link for hormones in the puzzle of HS pathophysiology.

### 2.2 X chromosome dosage

Another contributing factor to the female sex bias in autoimmune disorders is X chromosome dosage (30). Biologic females have two X chromosomes, and one of the X chromosomes undergoes inactivation (X chromosome inactivation; XCI) in early development (19). Patients with multiple X chromosomes are at increased risk of developing autoimmune disorders, such as SLE and SS, while these disorders have rarely been reported in patients with Turner’s syndrome, which is an X monosomy (14). The X chromosome harbors many genes involved in immune function, and XCI is often incomplete, with ~15% of genes escaping inactivation (14, 19). Among these are toll-like receptor (TLR)7 and TLR8, which are pattern recognition receptors that principally recognize endosomal single-stranded RNA. Activation of TLR7/8 induces expression of the inflammatory cytokines IL12, IL18, IL27, TNFα, and type I IFNs. Increased expression of TLRs may also contribute to the female sex bias in SLE, SSc, and SS through induction of a type 1 IFN response (14). Similarly, incomplete XCI and abnormal X chromosome dosage may contribute to the chronic inflammation observed in HS.

Furthermore, XCI is maintained by Xist/XIST RNA and heterochromatin modifications (e.g., DNA methylation and histone modifications) in immune cells (14). These processes contribute to the maintenance of appropriate X-linked gene dosage (14). For example, female-biased hypomethylation of the *CD40LG* promoter in T cells, resulting in greater *CD40LG* expression, is associated with SLE (14). CD40LG is a surface protein found on CD4+ T cells that binds to CD40 on antigen presenting cells, activating them to initiate a pro-inflammatory immune response (14). Similar heterochromatic perturbations may influence the expression of sex-biased genes that modulate immunity in patients with HS.

### 2.3 Other sex-biased genes

Additionally, there are sex-biased genes involved in immune function that are neither hormonally regulated nor found on the X-chromosome, such as *VGLL3 (* 13, 19 *).* VGLL3 is a transcription cofactor enriched in female skin that activates a gene regulatory network leading to the upregulation of proinflammatory and IFN-response genes (14, 31). Genes regulated by VGLL3 in female-predominant autoimmune disorders include B cell-activating factor (upregulated and therapeutically targeted in SLE), MMP9 (SLE, SSc, SS), IL7 (SLE, SS), and ICAM-1 (SLE) (31, 32). It is plausible that additional sex-biased transcription factors or proinflammatory gene modulators involved in autoinflammatory pathways exist and play a role in HS. Additional studies will help to clarify the molecular mechanisms of non-hormone and non-sex chromosome–related sex-biased gene expression.

## 3 Extrinsic immunological contributions to the sex bias in hidradenitis suppurativa

### 3.1 Microbiome

Increasing numbers of studies implicate a role for the microbiome in sex-biased immunity (19). Several studies have identified sex-related alterations in the cutaneous microbiome, which may result from differences between the sexes in hormone metabolism, rates of perspiration, and skin pH (33, 34). For example, sex-related differences in sebum production by the skin and its ability to retain moisture likely contribute to the differing levels of *Cutibacterium* (formerly known as *Propionibacterium)* on the skin of males and females (34). Notably, the cutaneous microbiome is altered in HS skin versus skin from healthy controls (4, 35). Furthermore, differences in the immune system such as those described above may influence the ways in which females respond to changes in the microbiome, which could in turn feedback and influence the microbiome yet further. Although it remains unknown whether sex-related changes in the skin microbiome contribute to HS, dysregulation of the skin microbiome within the context of female-biased immunity may promote Th1 and/or Th17-associated inflammation (4). Similar to the role of AMPs in the pathogenesis of acne, variation in cutaneous microbes in patients with HS may also directly affect production of AMPs by keratinocytes, contributing to a chronic inflammatory state in HS (36, 37).

The gut microbiome is also involved in both innate and adaptive immunity, and sex-related differences have been previously described (19, 38). For instance, sex-related alterations in the gut microbiota have been implicated in Crohn’s disease pathogenesis (39). Alterations in the gut microbiome have been reported in other inflammatory skin diseases as well, including psoriasis (40). Since HS has been consistently linked to diet, obesity, and metabolic syndrome, it is plausible that the gut microbiome influences disease presentation (41–43). Limited small-scale studies have identified changes in gut microbiome composition in patients with HS relative to controls. Larger studies are necessary to establish whether sex-related immunity associated with alterations in the gut microbiome may play a role in HS (41, 44). Furthermore, additional studies are required to explore the gut-skin axis and determine whether alterations in the gut microbiome may affect the cutaneous inflammation seen in HS.

### 3.2 Smoking

In contrast to the dramatic female sex bias in HS revealed in epidemiological studies from North America and Europe, several studies from Asia have shown a male sex bias of ~1:2 (F:M) (12, 45–47). The opposite trend likely represents a significant role of environmental influences on HS within the context of ancestry-specific genomic features. Smoking is one of the most substantiated environmental risk factors for HS (42). In a retrospective study conducted in Korea that included 438 patients with HS, the authors reported a higher proportion of male smokers than female smokers (47). In patients with HS, smoking has been associated with elevated leukocyte and neutrophil levels (48). Additionally, many chemicals found in tobacco smoke influence aryl hydrocarbon signaling (49). Recent work identified a potential pathophysiological link between aryl hydrocarbon receptor signaling and chronic inflammatory cutaneous disorders, such as HS (50, 51). Furthermore, benzopyrene, an aromatic compound found in cigarette smoke, contributes to increased levels of Th17 cells and higher IL17 expression, which are also findings seen in HS (36). Such changes in the response to cigarette-induced lung inflammation may also inadvertently impact the skin (Dimitrion et al., under review). In addition to inflammation associated with smoking, chemicals like nicotine, which are structurally related to endogenous molecules like NAD^+^, may perturb skin metabolism (52). Given recent findings that implicate CD38, an enzyme that degrades NAD+ into nicotinamide, in HS pathogenesis (Dimitrion et al., under review), future studies examining the downstream signaling pathways influenced by the presence and absence of nicotine may provide additional context into how smoking may directly influence immune cells in patients with HS.

## 4 Conclusion

HS is a debilitating inflammatory disease that demonstrates a strong female sex bias in numerous studies. The female sex bias in HS is likely multifactorial, with contributions from hormones, genetics, environmental influences, and the microbiome on changes in the immune system. Despite this, many previous studies examining the immunological profiles of patients with HS have not reported stratified results between biologic females and males. Furthermore, sex differences in the response to immunomodulatory HS biologics, such as adalimumab, have not studied (53, 54). It will be important to consider sex biased efficacy of immunomodulatory drugs in future trials in patients with HS considering the many potential sex-biased immunological contributions to HS. It is also interesting to note that differences in sex-biased immune processes may explain some of the growing literature supporting sex-biased co-morbidities associated with HS. However, in some of the comorbidities, the sex difference was not significant, such as psoriasis (55). A better understanding of sex-specific effects may also help reframe and improve management of HS in a more effective and equitable manner.

## Author contributions

Q-SM and IA conceived and designed the project. KY, Q-SM, and PD performed the literature review and drafted the manuscript. Q-SM, LZ and IA edited the manuscript. All authors contributed to the article and approved the submitted version.
